# Phosphorylation-dependent regulation of serine/arginine-rich proteins and U2AF1 interactions in early spliceosome assembly

**DOI:** 10.1016/j.jbc.2026.111222

**Published:** 2026-02-02

**Authors:** Zihan Zhang, Puspa Kunwar, Yanbao Yu, Peter Prevelige, Jun Zhang

**Affiliations:** 1 Department of Chemistry, University of Alabama at Birmingham, Birmingham, Alabama, USA; 2 Department of Chemistry and Biochemistry, University of Delaware, Newark, Delaware, USA; 3 Department of Microbiology, University of Alabama at Birmingham, Birmingham, Alabama, USA

**Keywords:** U2AF1, U2AF35, SRSF1, RNA splicing, phosphorylation

## Abstract

Early-stage spliceosome assembly is critical to constitutive and alternative pre-mRNA splicing. This process is orchestrated by serine/arginine-rich (SR) proteins (SRSF1–SRSF12) and SR-related proteins U1-70K and U2AF1. SR proteins recognize exonic splicing enhancers and interact with U1-70K and U2AF1 to recruit the U1 and U2 snRNP complexes to the 5′ and 3′ splice sites, respectively. However, the molecular basis of the interaction between SR proteins and U2AF1 has remained poorly understood, largely due to the poor solubility of full-length U2AF1. Here, we successfully refold and solubilize U2AF1 and confirm its structural integrity. This enables investigation of its interaction with SRSF1, a prototypical SR protein. We show that the U2AF1 C-terminal RS domain (RS^U2AF1^) is essential for binding to the phosphorylated RS domain of SRSF1 (RS^SRSF1^), and that RS^U2AF1^ is phosphorylated in cells. Notably, phosphorylation of RS^U2AF1^ significantly reduces its affinity for SRSF1, revealing a phosphorylation-dependent regulatory mechanism. The SRSF1–U2AF1 interaction closely parallels that of SRSF1 and U1-70K, hinting at a general principle in which phosphorylated RS interacts with unphosphorylated ones. Inspired by this discovery, we further find the interaction between phosphorylated and unphosphorylated SRSF1, providing a mechanistic explanation of long observed self-interactions within SR proteins. Our molecular dynamics simulations further reveal that the salt-bridges between phosphoserine and arginine dominate these interactions, and the interaction strength depends on net charges of RS regions. Together, our findings provide new molecular insights into how phosphorylation modulates splicing factor interactions and highlight a conserved mechanism that regulates early spliceosome assembly.

Eukaryotic gene expression relies on the precise processing of precursor messenger RNA (pre-mRNA) to generate mature mRNA transcripts. A critical step in this process is pre-mRNA splicing, which removes noncoding introns and joins coding exons to produce functional mRNAs for translation. This process is essential for generating protein diversity and regulating gene expression in response to developmental and environmental cues (1, 2). Defects in splicing regulation have been implicated in numerous human diseases, including cancer and neurodegenerative disorders, highlighting the importance of understanding the molecular mechanisms governing splicing (3, 4).

Pre-mRNA splicing is catalyzed by the spliceosome, a dynamic and complex machinery composed of small nuclear ribonucleoproteins (U1, U2, U4, U5, and U6 snRNPs) and associated protein factors (5, 6, 7). Due to sequence degeneracy of the splicing sites, snRNPs alone are unable to ensure authentic splicing. Instead, various splicing factors are needed to guide and regulate the spliceosome (8). Ser/Arg-rich splicing factors (SR proteins) are a dominant family of splicing factors comprised of 12 members, SRSF1 to SRSF12 (9, 10). SR proteins consist of one to two RNA-recognition motifs (RRMs) and an arginine-serine–rich (RS) region (11, 12, 13). SR proteins primarily recognize exonic splicing enhancer in pre-mRNA transcripts through their RRM domains. While the RS domains are subject to different extent of phosphorylation and mediate protein–protein interactions (8, 14, 15, 16, 17, 18, 19).

The interaction of SR proteins with U1-70K and U2AF1 (formerly U2AF35) recruits U1 and U2 snRNP to the 5′ and 3′ splicing sites, respectively (20, 21, 22). These protein–protein interactions initiate the early stage spliceosome assembly and determine exon selection and therefore are of paramount importance for alternative splicing (23, 24, 25, 26, 27). Our recent study has elucidated the interaction of U1-70K and SRSF1 at the structural level (28). The interaction of U2AF1 with SRSF1 (SF2/ASF) and SRSF2 (SC35) has been confirmed by yeast two-hybrid and in-cell FRET studies (19). However, due to the poor solubility of U2AF1 and SRSF1 (29), the molecular determinants of the interaction between SR proteins and U2AF1 are poorly understood.

U2AF1 forms a stable complex with U2AF2 (30). U2AF1 specifically interacts with the conserved AG dinucleotide at the intron–exon junction, and U2AF2 binds to the polypyrimidine tract upstream of the 3′ splice site (9, 31). In line with its central role in RNA splicing, U2AF1 is the most frequently mutated protein in myelodysplastic syndromes and related disorders (32). U2AF1 is a structurally unique RNA-binding protein containing an RRM flanked by two zinc fingers and an RS region (Fig. 1*A*). To distinguish the RS domains of U2AF1 and SRSF1, they are named RS^U2AF1^ and RS^SRSF1^ in this study, respectively.

**Figure 1:**
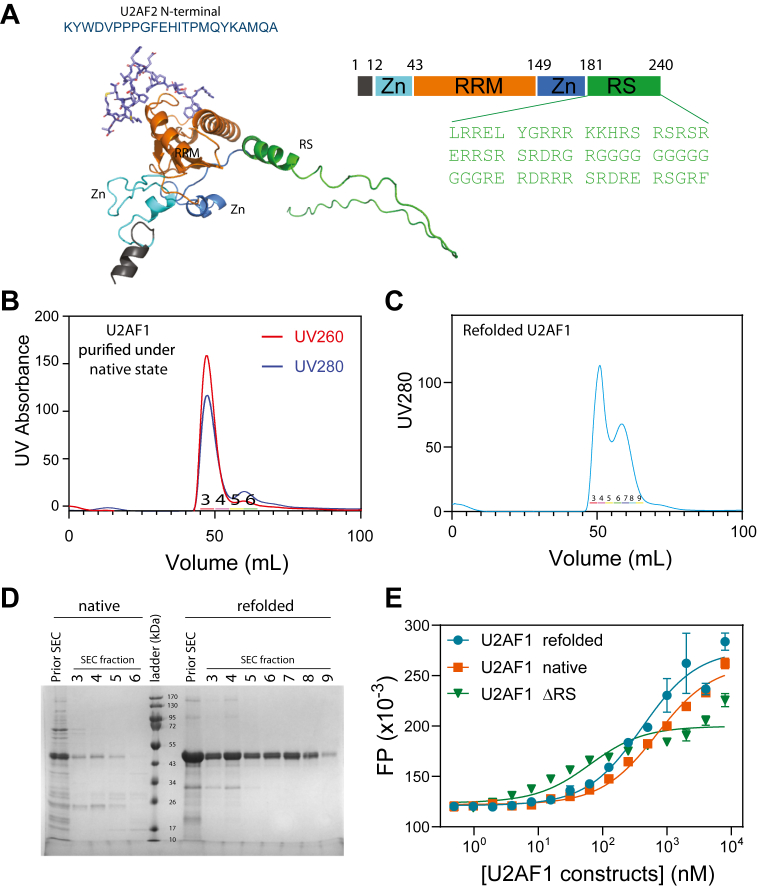
**Purification and refolding of human U2AF1.***A*, domain architecture and AlphaFold-predicted structure of U2AF1. The structural cartoon uses the same color scheme as the domain schematic shown above. The U2AF2 peptide (shown as *sticks*) was modeled based on the crystal structure (PDB ID: 1JMT). The amino acid sequence of the arginine/serine-rich (RS) tail is shown below. *B*, size-exclusion chromatography (SEC) profile of U2AF1 purified under the native state. *C*, SEC profile of refolded U2AF1 purified under the denatured state. *D,* the SDS-PAGE gel for the SEC fractions shown in *panel B* and *C*. *E*, refolded U2AF1 exhibits a similar binding affinity to the U2AF2 peptide as U2AF1 purified under native conditions. The *error bars* indicate standard deviations from three duplicated experiments. RRM, RNA-recognition motif.

We resolve the solubility issue of U2AF1, which provides us with an opportunity to elucidate the molecular determinants of U2AF1-SRSF1 interaction. We show that U2AF1 is challenging to purify in the native state, necessitating a refolding approach to obtain functionally active protein. The success of U2AF1 refolding is confirmed by its binding with U2AF65. We further show that the zinc fingers of U2AF1 are essential for its structural stability, while its RS domain mediates the interaction with phosphorylated SRSF1. We find that the U2AF1 RS region is phosphorylated in cells and can be phosphorylated *in vitro* by CLK1 and SRPK1. Fluorescence polarization (FP) and pull-down assays reveal that phosphorylation of the U2AF1 RS domain weakens its binding to SRSF1, suggesting a potential regulatory mechanism. This is reminiscent of the interaction between SRSF1 and U1-70K, which is also mediated by phosphorylated RS^SRSF1^ and unphosphorylated RS^U1-70K^ (28), hinting at a general principle in which phosphorylated RS regions interact with unphosphorylated ones. Inspired by this finding, we found that phosphorylated SRSF1 interacts with unphosphorylated SRSF1, in line with the long-observed self-interactions within SR proteins. We further perform molecular dynamics (MD) simulation and find that salt bridges between phosphoserine and arginine residue dominate the RS-mediated interactions, followed by H-bonds and stacking interactions. By comparing the complexes of phosphorylated RS^SRSF1^, we find that the numbers of interactions are ranked as basic-acidic dipeptide domain 1 (BAD1): pi-RS^SRSF1^ > RS^U2AF1^: pi-RS^SRSF1^ > RS^SRSF1^: pi-RS^SRSF1^, consistent with experimentally measured binding affinities (28). Collectively, these findings provide new insights into the biochemical properties of U2AF1 and its role in splicing regulation through interactions with SR proteins and the general principle for interactions mediated by RS regions.

## Results

### Purification and refolding of U2AF1

Human U2AF1 contains an RRM flanked by two zinc fingers and a low-complexity region rich in RS residues (Fig. 1*A*). However, obtaining sufficient soluble, full-length U2AF1 is challenging due to its poor insolubility. Most of the recombinant protein accumulates in inclusion bodies, with only a small fraction remaining in the soluble fraction. Moreover, U2AF1 purified under native conditions contains degradation products and nucleic acid contaminants that cannot be removed by size-exclusion chromatography (SEC), resulting in poor yield and purity (Fig. 1*B*).

To address these issues, we adopted a denaturation–refolding strategy. Cells were lysed under denaturing conditions, and U2AF1 was refolded directly on Ni-NTA resin by gradually reducing guanidinium concentrations. Given its low solubility in PBS—similar to that of other arginine-rich proteins such as SRSF1 and U1-70K—we dissolved the refolded protein in a buffer containing 400 mM Arg/Glu (Fig. 1*C*). It turned out that the refolded U2AF1 had a higher purity compared with the protein purified under the native state (Fig. 1*D*).

To validate proper refolding, we performed FP assays to measure binding of U2AF1 to the N-terminal peptide of U2AF2 (30). The refolded protein exhibited binding affinity comparable to that of the native-purified sample, with a slightly higher affinity (400 ± 100 nM *versus* 690 ± 90 nM), likely due to reduced contamination (Fig. 1*E*). Notably, deletion of the RS tail significantly enhanced binding affinity (50 ± 30 nM), suggesting an autoinhibitory role of the RS domain. In summary, we successfully refolded and solubilized full-length human U2AF1, enabling downstream biochemical characterization.

### Flanking zinc fingers contribute to the structural stability of U2AF1

**Figure 2:**
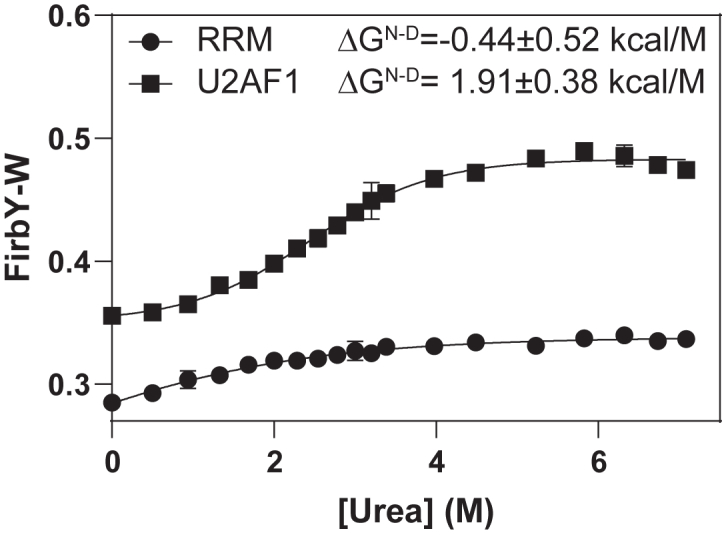
**Zinc fingers are important for stability of U2AF1 RRM.** ΔG^N-D^ represents the Gibbs free energy difference between the native and the denatured states. Values were determined from urea-induced unfolding curves using the FirbY-W FRET-based assay. The *error bars* indicate standard deviations from two duplicated experiments. RRM, RNA-recognition motif.

The RRM of U2AF1 is atypical in that it is flanked by two zinc fingers, which are known to contribute to RNA binding. Structural analysis reveals that several hydrophobic residues (I24, L109, Y114, V147, F150, and L174) are packed at the interface between the RRM and zinc fingers, suggesting a stabilizing interaction. To investigate the role of these zinc fingers in protein stability, we employed FirbY-W, a FRET-based assay that monitors protein unfolding by measuring the fluorescence intensity ratio of tyrosine to tryptophan (33) (Fig. 2). Protein unfolding is indicated by an increase in the Tyr/Trp ratio. Deletion of individual zinc finger results in poorly behaved proteins, which are unsuitable for further characterization. Deletion of both zinc fingers significantly destabilized U2AF1, with the free energy of unfolding decreasing from 1.91 kcal/mol (wildtype) to −0.44 kcal/mol (zinc finger deletion), indicating that the zinc fingers are essential for maintaining the structural integrity of U2AF1.

### U2AF1 undergoes reversible oligomerization

**Figure 3:**
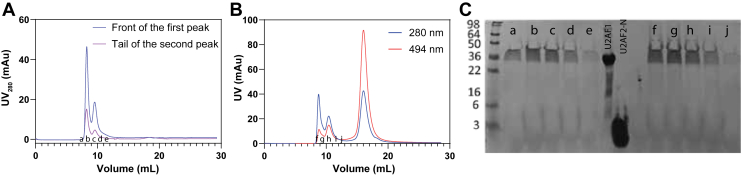
**U2AF1 undergoes reversible oligomerization and both species bind U2AF2 peptide.***A*, size-exclusion chromatography (SEC) of U2AF1 on a Superdex Increase 75 10/300 Gl column. The leading edge of the large-species peak and the trailing edge of the small-species peak were individually collected and re-injected. Both samples resolved into two peaks again, indicating reversible oligomerization. *B,* SEC analysis of U2AF1 in complex with a fluorescein-labeled U2AF2 N-terminal peptide. Absorbance at 495 nm confirms that both oligomeric species are capable of binding the peptide, suggesting that both are folded and functionally active. *C*, the SDS-PAGE image of the SEC fractions shown in *panel A* and *B*. The purified full-length U2AF1 and U2AF2 N-terminal peptide (U2AF2-N) were included in the gel as controls. The SEC fractions and their corresponding lanes SDS-PAGE are denoted by letter from a to j.

As shown in Figure 1*C*, U2AF1 exhibits oligomerization on SEC, and two peaks were observed, referred to as large and small species. We first wanted to test whether the oligomerization of U2AF1 is reversible. To this end, we collected the peak front of the first peak from SEC and reloaded it onto SEC (Fig. 3*A*). According to the profile deconvolution, the peak front contained negligible amounts of small species. However, we found that two species were observed again. We also collected the tail of the small species peak and reloaded it onto SEC. As expected, we also observed the large species developed during the elution (Fig. 3*A*). In addition, the ratio of the large to small species increases along with the concentration of analyte. All these results suggest that U2AF1 oligomerization is reversible. We also resolved the U2AF1 protein bound to fluorescein-labeled U2AF2 peptide, which absorbs at 495 nm (Fig. 3*B*). We also confirmed that SEC fractions from reloaded samples are U2AF1 by SDS-PAGE (Fig. 3*C*). The SEC profile revealed that both the large and the small species bound with the U2AF2 peptide, suggesting both species are folded in the native state. We also observed that the larger species bound more U2AF2 peptide than the smaller one. Because the complex structure (Fig. 1*A*) shows a 1:1 stoichiometry, this difference likely results from partial dissociation of the complex during SEC elution: the larger species is more prone to separation from the peptide, suggesting that the U2AF1–U2AF2 heterodimer is reversible.

### U2AF1 is phosphorylated in cells and is a substrate of SRPK1 in vitro

U2AF1 is an SR-related protein characterized by two RS regions separated by a glycine-rich linker. Our previous work demonstrated that the RS domain of another SR-related protein, U1-70K, was phosphorylated in the BAD1 by SRPK1. To investigate whether U2AF1 is similarly phosphorylated, we examined its phosphorylation status both in the cell and *in vitro*.

**Figure 4:**
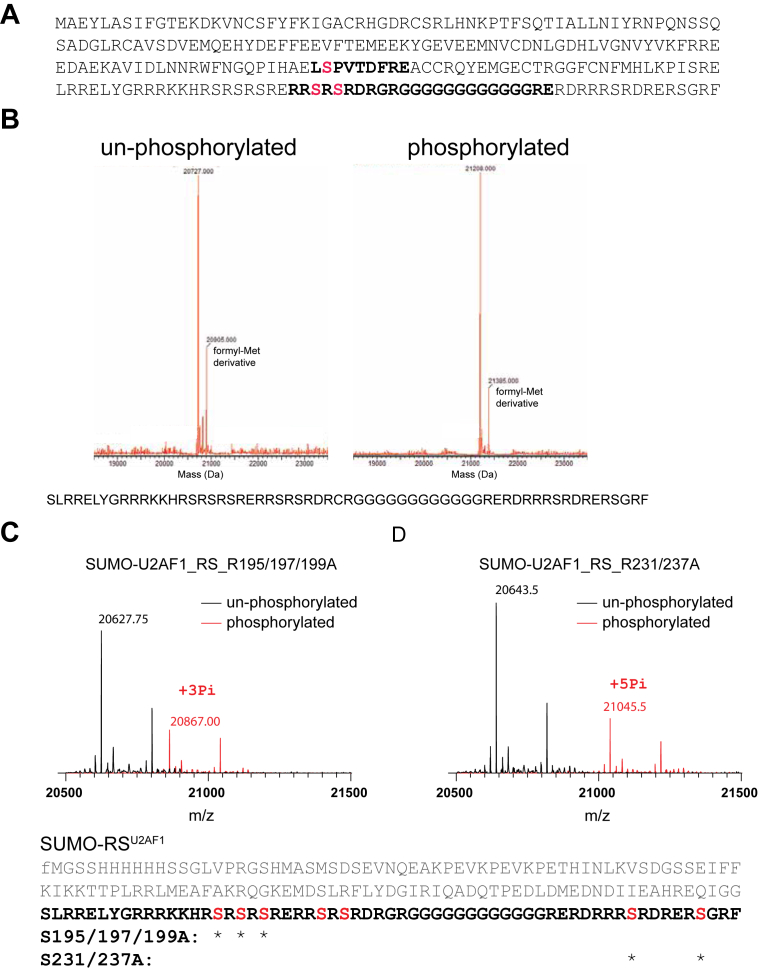
**U2AF1 is phosphorylated in cells and can be phosphorylated by SRPK1 *in vitro*.***A*, U2AF1 purified from HEK293-FT cells was digested with Glu-C and analyzed by mass spectrometry. Two phosphorylation sites were identified within the RS region. Detailed results are shown in Table S1. *B*, *in vitro* phosphorylation assays with SRPK1 demonstrated that SRPK1 adds up to six phosphate groups to the U2AF1 RS domain. *C,D,* intact mass spectrometry analysis of SUMO-tagged U2AF1 RS mutants: (*C*) R195/197/199A and (*D*) R231/237A. The protein sequence of SUMO-tagged (*gray*) RS^U2AF1^ (*black bold*) is shown below, with serine residues in RS repeats highlighted in *red*. “∗” denotes sites of mutation. Note that the N-terminal methionine is formylated (fM) and is frequently removed during expression in *E. coli*. RS, arginine-serine–rich.

His-tagged U2AF1 was purified from 293FT cells and analyzed by mass spectrometry (MS) (Table S1). MS analysis identified two phosphorylation sites on the RS domain of U2AF1 (Fig. 4*A*). However, it is well known that phosphorylated RS regions are challenging to detect by MS due to their high charge and low ionization efficiency (28, 34); therefore, this likely underestimates the extent of phosphorylation in the cell. To assess whether SRPK1 can phosphorylate U2AF1 directly, we performed *in vitro* kinase assays and found that SRPK1 phosphorylated six of seven serine residues in U2AF1 RS, indicating that U2AF1 is a *bona fide* substrate of SRPK1 (Fig. 4*B*). SRPK1 specifically recognizes and phosphorylates Arg–Ser (RS) repeat motifs (35, 36). In U2AF1, RS repeats are clustered near both the N-terminal region (residues 195–205) and the C-terminal region (Ser231 and Ser237). We initially attempted to identify phosphorylation sites directly by MS; however, despite testing multiple proteases, no phosphorylated RS-containing peptides were detected. This limitation is well documented for highly phosphorylated RS regions and was also encountered in our previous analysis of the BAD1 region of U1-70K (28). To circumvent this issue, we individually mutated the two RS clusters (Fig. 4, *C* and *D*). In the S195/197/199A mutant, three of the four remaining serine residues were phosphorylated, indicating that at least one serine within the C-terminal RS cluster can serve as an SRPK1 phosphorylation site. Conversely, in the S231/237A mutant, all five serine residues in the N-terminal RS cluster remained phosphorylated. Collectively, these results demonstrate that RS repeats at both the N- and C-terminal regions of U2AF1 are substrates for SRPK1-mediated phosphorylation.

### RS^U2AF1^ is responsible for interaction with phosphorylated SRSF1

**Figure 5:**
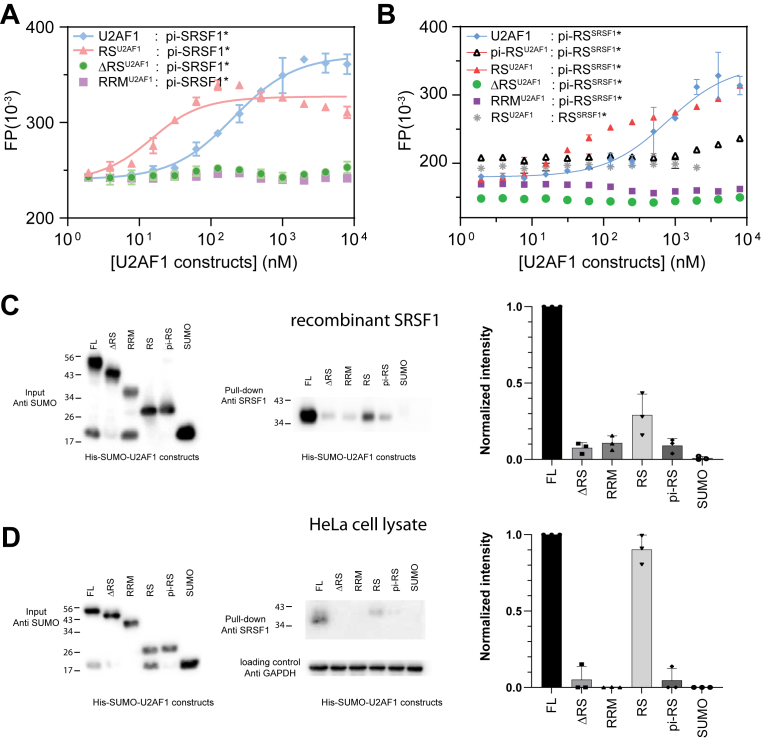
**The RS tail of U2AF1 mediates its interaction with phosphorylated SRSF1 (pi-SRSF1).***A*, fluorescence polarization (FP) assays showing the binding between pi-SRSF1 and various U2AF1 constructs. *B*, FP assays measuring binding between a phosphorylated RS tail peptide of SRSF1 and the same U2AF1 constructs. For both SRSF1 constructs, Alexa Fluor 488 was conjugated at the engineered C-terminal cysteine (T248C). The concentration of the fluorescent probe was 10 nM in all assays. Fluorescence-labeled molecules are denoted by ∗. Standard deviations were estimated from three replicate measurements. *C*, His-SUMO tagged U2AF1 were immobilized on Ni-NTA bead as baits. Hyperphosphorylated SRSF1 purified from *E. coli* was used as the prey. Sumo-specific and SRSF1-specific antibodies were used to detect the input U2AF1 and pull-down SRSF1. The *p*-values for comparisons between FL and each of the other constructs are <0.001. *D*, HeLa cell lysate was used as the prey. GAPDH-specific antibody was used to ensure similar amounts of cells were used. The *p*-value for FL and RS is 0.1489, and the *p*-values for FL and the rest constructs are <0.0001. All pull-down assays were performed three times, and the standard deviation was estimated from these replicates. RS, arginine-serine–rich; RRM, RNA-recognition motif.

To identify the U2AF1 domains critical for interacting with SRSF1, we measured the binding affinity of fluorescently labeled, phosphorylated SRSF1 to various U2AF1 constructs using FP. Full-length U2AF1 bound phosphorylated SRSF1 with an affinity of ∼200 nM, while the isolated RRM^U2AF1^ domain showed no detectable binding (Fig. 5 and Table 1). Deletion of RS^U2AF1^ also abolished interaction, indicating that it is essential for SRSF1 binding. Interestingly, RS^U2AF1^ exhibited a markedly higher affinity (∼9 nM), suggesting that other regions of U2AF1 may exert an inhibitory effect on SRSF1 binding (Fig. 5*A*).

**Table 1:** Dissociation constants of U2AF1 and SRSF1 constructs

Fluorophore-labeled protein (10 nM)	Titrant	Kd (nM)
pi-SRSF1	U2AF1	200 ± 21
pi-SRSF1	RS^U2AF1^	9 ± 2.6
pi-SRSF1	ΔRS^U2AF1^	Too weak to be determined
pi-SRSF1	RRM^U2AF1^	Too weak to be determined
pi-RS^SRSF1^	U2AF1	780 ± 160
pi-RS^SRSF1^	RS^U2AF1^	Too weak to be determined
pi-RS^SRSF1^	RS^U2AF1^	60 ± 9.6
pi-RS^SRSF1^	ΔRS^U2AF1^	Too weak to be determined
pi-RS^SRSF1^	RRM^U2AF1^	Too weak to be determined
RS^SRSF1^	ΔRS^U2AF1^	Too weak to be determined

Dissociation constants data for Figure 5.

We next tested the role of RS^SRSF1^: the phosphorylated RS domain of SRSF1 bound full-length U2AF1 with a weaker affinity (∼780 ± 160 nM) compared to full-length SRSF1, suggesting that although the phosphorylated RS domain is critical, the RRMs of SRSF1 also contribute to the interaction (Fig. 5*B*, Table 1). Notably, phosphorylated SRSF1 RS bound the U2AF1 RS domain more tightly than full-length U2AF1 (∼60 ± 9.6 nM *versus* 780 ± 160 nM, Table 1), consistent with prior evidence that RS^SRSF1^ engages in intramolecular interactions with its RRM tandem, which may partially occlude binding sites (37).

To validate the FP results, we performed pull-down assays using purified recombinant SRSF1 (Figs. 5*C*, S1, *A* and *B*) and HeLa cell lysates (Figs. 5*D*, Fig. S1, *C* and *D*). Full-length U2AF1 and RS^U2AF1^ pulled down phosphorylated SRSF1, while RRM^U2AF1^ did not. Phosphorylation of RS^U2AF1^ (pi-RS) weakened SRSF1 binding, likely due to increased electrostatic repulsion. Similar trends were observed in the HeLa cell lysate, though bands were weaker due to lower endogenous SRSF1 abundance (Fig. 5*D*).

### RS^SRSF1^ interacts with U2AF1

**Figure 6:**
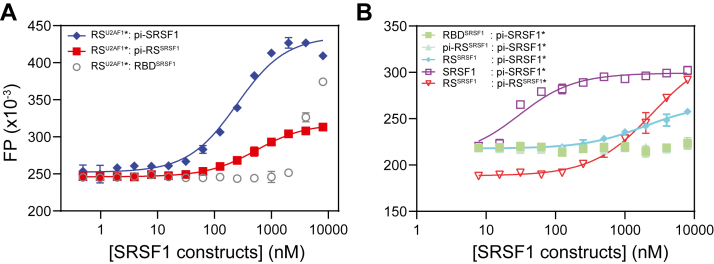
**Binding between phosphorylated RS and unphosphorylated RS.***A*, phosphorylated SRSF1 RS (pi-SRSF1 RS) binds to the RS tail of U2AF1. Fluorescence polarization (FP) assays were performed using His-SUMO–tagged U2AF1 RS domain labeled at F240C with Alexa Fluor 488 (10 nM). Assays were conducted in PBS supplemented with 0.1 mM TCEP and 0.02% Tween-20. *B*, phosphorylated SRSF1 or SRSF1 RS binds with unphosphorylated SRSF1 or SRSF1 RS. Assays were conducted in 20 mM Tris-HCl pH 8.5, 200 mM Arg/Glu, 0.1 mM TCEP, and 0.02% Tween-20. Alexa Fluor 488 was labeled at T248 C for pi-SRSF1 or pi-RS^SRSF1^. In *panel A* and *B*, fluorescence-labeled molecules are denoted by ∗. Standard deviations were estimated from three replicate measurements. RS, arginine-serine–rich.

We have mapped the SRSF1-interacting region to the RS domain of U2AF1. To identify the SRSF1 domains responsible for this interaction, we labeled the U2AF1 RS domain at F240 C with Alexa Fluor 488. As shown in Figure 6*A*, FP assays revealed that the phosphorylated RS^SRSF1^ binds to RS^U2AF1^ with a dissociation constant of approximately 500 nM (Table 2). In contrast, a much weaker interaction was observed between RS^U2AF1^ and the tandem RRM domains of SRSF1, suggesting that RS^SRSF1^ is the major contributor for the interaction. Notably, this binding affinity differs from that observed when Alexa488 is labeled on phosphorylated RS^SRSF1^ (Kd ∼60 nM), suggesting that fluorophore positioning influences apparent affinity (Fig. 6*A*, Table 2). This difference likely reflects the electrostatic properties of Alexa488, which carries two to three negative charges. When conjugated to phosphorylated RS^SRSF1^, the overall negative charge distribution remains consistent with the protein's phosphorylation state, resulting in minimal perturbation of binding to the electropositive RS^U2AF1^. In contrast, labeling Alexa488 on RS^U2AF1^ introduces a localized negative charge that likely repels the phosphorylated RS^SRSF1^, weakening the interaction. Given that the U2AF1–SRSF1 interaction is mediated by intrinsically disordered regions, fluorophore labeling is more sensitive to the local dynamics of the labeling site and less sensitive to global tumbling. As demonstrated previously, Alexa488 itself does not bind phosphorylated SRSF1 RS (28), further supporting that the observed interactions are specific and biologically meaningful.

**Table 2:** Dissociation constants between SRSF1 and RS^U2AF1^, SRSF1 and phosphorylated SRSF1

Fluorophore-labeled protein (10 nM)	Titrant	Kd (nM)
RS^U2AF1^	pi-SRSF1	240 ± 40
RS^U2AF1^	RBD^SRSF1^	Too weak to be determined
RS^U2AF1^	pi-RS^SRSF1^	530 ± 70
pi-SRSF1	RBD^SRSF1^	Too weak to be determined
pi-SRSF1	pi-RS^SRSF1^	Too weak to be determined
pi-SRSF1	RS^SRSF1^	1900 ± 380
pi-SRSF1	SRSF1	25 ± 4.0
pi-RS^SRSF1^	RS^SRSF1^	2500 ± 260

Dissociation constants data for Figure 6.

In this study, we found that phosphorylated RS^SRSF1^ interacts with RS^U2AF1^, which is reminiscent of the interaction between RS^SRSF1^ and the RS region in U1-70K (28). It seems that phosphorylated RS regions interact with unphosphorylated RS regions. To check whether this is generally applicable, we measured binding affinity between phosphorylated SRSF1 and unphosphorylated SRSF1 (Fig. 6*B*) and found a binding affinity with a Kd of 25 nM (Table 2). Neither the RNA-binding domain (the N-terminal RRM1 and RRM2, RBD^SRSF1^) or phosphorylated RS^SRSF1^ showed detectable interaction with phosphorylated SRSF1. However, unphosphorylated RS^SRSF1^ binds to phosphorylated SRSF1 with an affinity of 1900 ± 260 nM. Similar binding affinity was also detected between phosphorylated RS^SRSF1^ and unphosphorylated RS^SRSF1^. Together, these results suggest that RS^SRSF1^ is responsible for SRSF1 self-interaction, although RBD^SRSF1^ also contributes.

### A general rule of RS-mediated interactions: Phosphorylated RS interacts with unphosphorylated RS

**Figure 7:**
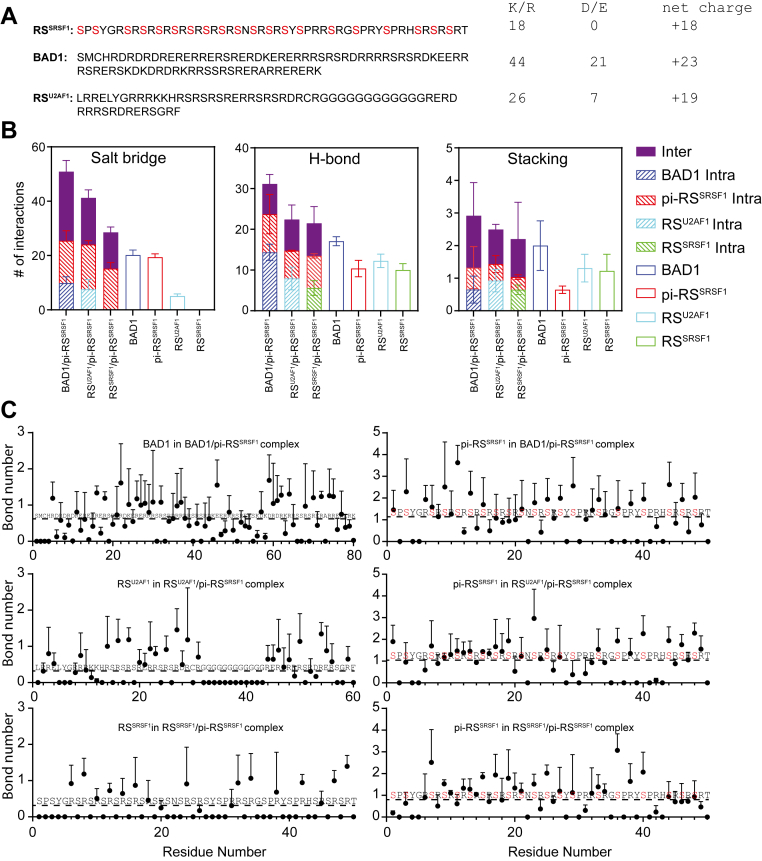
**Molecular dynamics analysis of interactions mediated by RS regions.***A*, the protein sequences of RS regions from SRSF1 (RS^SRSF1^), U1-70K (BAD1), and U2AF1 (RS^U2AF1^) were used in simulations. The number of basic and acidic amino acids and net charges are shown on the left. In RS^SRSF1^, phosphorylated serine residues are highlighted in *red*. *B*, bar graphs represent the average number of interactions from three replicate simulations starting at three different conformations per frame over a 150-ns trajectory following equilibrium. Salt bridges, hydrogen bonds, and stacking interactions were quantified. For complexes, interchain interactions are shown as *solid bars* and intrachain interactions as *hatched bars*. For unbound RS regions (*open bars*), total intramolecular interactions were quantified. Error bars indicate standard deviations across three replicate simulations starting at three different conformations. *C,* the average number of salt bridges formed for each residue per frame from three replicate simulations starting at three different conformations. The averages were calculated for 1000 frames. The *dash lines* indicate the average for all residues in corresponding peptide. RS, arginine-serine–rich; BAD1, basic-acidic dipeptide domain 1.

To gain atomic-level insight into RS-mediated interactions, we performed MD simulations and compared the complexes of phosphorylated SRSF1 RS formed with U1-70K BAD1, RS^U2AF1^, and unphosphorylated SRSF1 RS (RS^SRSF1^) (Fig. 7*A*). After the systems reached equilibrium (Fig. S2), we analyzed the intrachain and interchain salt bridges, H-bonds, and stacking interactions among 1000 frames that encompass 150 ns. The stacking interactions were searched among Arg and aromatic amino acids. It is still challenging to precisely calculate the free energy of a binding event involving disordered proteins. Therefore, we quantified the average number of interactions per frame to estimate the contribution of different types of interactions to the complexes. The number of salt bridges surpass that of H-bonds and stacking interactions. Given that salt bridges are generally more stable than H-bonds and stacking interactions, we concluded that salt bridges were the primary driving force for these RS-mediated complexes. More interestingly, these three types of interactions in these complexes followed an order of BAD1/pi-RS^SRSF1^ > RS^U2AF1^/pi-RS^SRSF1^ > RS^SRSF1^/pi-RS^SRSF1^, the same as the experimentally measured binding affinities. This coincidence verified our analytic approach to the system.

pi-RS^SRSF1^, BAD1, and RS^U2AF1^ contain both electropositive and electronegative residues. In their unbound state, extensive intrachain salt bridges were found between phosphoserine and Arg for pi-RS^SRSF1^, and between Arg and Asp/Glu for BAD1 or RS^U2AF1^ (Fig. 7*B*). However, the number of intrachain salt bridges within BAD1 or pi-RS^SRSF1^ were largely replaced by the interchain ones in the bound state. Replacement of intrachain interactions by interchain ones was also observed for H-bonds and stacking interactions, albeit to a less extent.

Since salt bridges were the main contributors for these RS-mediated complexes, we further analyzed the average salt-bridge number formed for each residue (Fig. 7*C*). As expected, in these three complexes, phosphoserine residues were the main salt-bridge acceptors in pi-RS^SRSF1^. Due to the repetitive feature, no outstanding hotspots could be identified for in these RS peptides. However, we found that the “RS” and consecutive R motifs in BAD1 demonstrated notable higher salt bridges compared with “RE” or “RD” motifs. This is likely due to overall net positive charges, which promotes interaction with electronegative pi-RS^SRSF1^. This analysis also explained our previous observation that BAD2, which contains more “RE” and “RD” motifs, binds to SRSF1 with a lower affinity.

## Discussion

The fidelity of mRNA splicing is controlled by snRNP and hundreds of splicing factors. A striking feature of these factors is that many of them contain RS regions. These proteins are called SR proteins and SR-related proteins. SR proteins contain 12 members and SR-related proteins represent an even larger protein family consisting of 40 to 50 members, such as U1-70K, U2AF1, U2AF2, and CLK kinases. RS regions of SR proteins have long been assumed to mainly mediate protein–protein interaction, but to what proteins and by what mechanism these regions bind to their binding patterns are unknown. The SRSF1 and SRSF2 (SC35) have been reported to interact with U2AF1 (19). However, due to challenges in obtaining soluble full-length U2AF1, it has been difficult to define the essential domains mediating these interactions. In this study, we successfully refolded U2AF1, enabling systematic analysis of domain contributions to both protein stability and the SRSF1–U2AF1 interaction.

We found that U2AF1 forms reversible oligomers, and all observed oligomeric species retain the ability to bind the N-terminal peptide of U2AF2. This oligomerization behavior is consistent with previous reports of U2AF1 self-association (19, 38). Interestingly, oligomerization does not depend on the RS tail, suggesting other domains mediate this oligomerization. The functional implications of U2AF1 oligomerization remain to be determined. Our SEC analysis (Fig. 3*B*) also hints at the reversibility of the U2AF1/U2AF2 heterodimer. This observation is consistent with a previous study showing that chromatin-crosslinked U2AF1 did not coprecipitate with U2AF2 (39). These findings imply that U2AF1 can exist independently and may perform functions beyond pre-mRNA splicing, such as translation regulation and ribosomal biogenesis (39, 40). The reversable nature of the U2AF1–U2AF2 interaction may thus represent an additional layer of regulation for splicing factors. Notably, U2AF1 binding has been shown to induce distinct conformations in U2AF2 (41), further supporting the dynamic and regulated nature of their association.

Our results demonstrate that the zinc fingers are important for U2AF1 stability and that the U2AF1 RS domain is essential for SRSF1 interaction. Interestingly, the myelodysplastic syndromes-associated mutations (S34/Q157) are located within the two zinc fingers. Although these mutations have been shown to alter the RNA-binding specificity of U2AF1 (41, 42, 43), they may also impact protein stability. The essential role of RS in SRSF1 binding parallels our previous study on the SRSF1: U1-70K complex, where phosphorylated SRSF1 RS interacts with the BAD1 domain of U1-70K (28). In both cases, phosphorylation of the SRSF1 RS domain is critical for binding. This observation makes biological sense as recruitment of U1 and U2 by SR proteins occurs sequentially. We discovered that the U2AF1 RS domain contains at least two phosphorylation sites in the cell. Given that hyperphosphorylated RS peptides (such as BAD1) tend to aggregate and yield weak MS signals (28), additional phosphorylated regions in U2AF1 RS may escape detection. SRPK1 or SRPK2 could be the kinases responsible for U2AF1 phosphorylation, as these two kinases have been found to interact with U2AF1 by multiple studies (44, 45, 46, 47, 48). In addition, recent studies have found that mTOR regulates the isoform balance of U2AF1 and activates SRPK2, which phosphorylates SRSF1 (49, 50). Collectively, these studies support a central role of phosphorylation in regulating splicing factor activity.

Our previous work showed that phosphorylated SRSF1 RS binds U1-70K BAD1 through the RS repeats, while phosphorylation of BAD1 itself diminishes its interaction with SRSF1 (28). In this study, we find a similar regulatory mechanism for U1AF1 and SRSF1. In line with this, we also find that phosphorylated SRSF1 or its RS domain interacts with its unphosphorylated counterpart, suggesting a broader principle: phosphorylated RS domains preferentially interact with unphosphorylated RS domains. Our findings help explain the reported self-interactions of SR proteins or interactions between SR proteins—potentially arising from heterophosphorylation states within the RS domain family (19). Such differential phosphorylation could serve as a regulatory mechanism for modulating SR protein interactions. Our MD simulations suggested that phosphoserine residues of pi-RS^SRSF1^ are a key player by interacting arginine residues of BAD1 or RS^U2AF1^. Phosphorylation regulates these interactions in two ways. Phosphorylation of BAD1 or RS^U2AF1^ reduce their net positive charges and weaken their attractive interaction with pi-RS^SRSF1^. Moreover, as found in this and recent studies (37), phosphoserine residues in RS regions tend to be engaged in interactions with adjacent arginine. That means that a phosphorylated RS has a higher tendency for intrachain interaction compared with unphosphorylated RS. These two effects reduce the interactions of pi-RS^SRSF1^ with BAD1 or RS^U2AF1^. Considering that many SR proteins and SR-related proteins contain RS regions and participate in RNA splicing, it is still an intriguing question how phosphorylation of these proteins is coordinated in the cell.

## Experimental procedures

### Purification and refolding of U2AF1

The DNA sequence encoding human U2AF1 was cloned into the pSMT3 vector downstream of an N-terminal His_6_-SUMO tag. Constructs were expressed in *E. coli* BL21-CodonPlus (DE3) cells cultured in LB medium supplemented with 50 μg/ml of kanamycin and 50 μg/ml of chloramphenicol. Protein expression was induced with 0.5 mM IPTG at an absorbance (*A*_600_) of 0.6, followed by overnight incubation at 22 °C. Cells were harvested and resuspended in lysis buffer containing 6 M guanidinium-HCl, 2 M NaCl, 20 mM Tris-HCl (pH 7.5), 25 mM imidazole, 0.2 mM TCEP, and 0.5 mg/ml lysozyme, then sonicated in an ice-water bath.

The lysate was clarified by centrifugation at 23,710*g* for 40 min at 4 °C. The supernatant was applied to a 5 ml Ni-NTA resin column. Refolding and contaminant removal were achieved through stepwise washes (50 ml each) using refolding buffer containing 4.8 M, 3.6 M, 2.4 M, 1.2 M, and 0 M guanidinium-HCl in 20 mM Tris-HCl (pH 7.5), 2 M NaCl, 25 mM imidazole, 0.2 mM TCEP, and 1 μM ZnCl_2_. The His_6_-SUMO-U2AF1 fusion protein was eluted using 30 ml of 20 mM MES (pH 6.0), 0.5 M arginine/glutamate, 0.5 M imidazole, and 0.2 mM TCEP. Eluted protein was concentrated and further purified by SEC using a Superdex 75 pg 16/600 column (Cytiva) equilibrated in 400 mM Arg/Glu (pH 6.0) with 0.2 mM TCEP. Purity was confirmed by SDS-PAGE.

### FP binding assays

To verify correct refolding of His_6_-SUMO-U2AF1, FP binding assays were performed using a 5-FAM-labeled U2AF2 ULM peptide (5-FAM-KKKVRKYWDVPPPGFEHITPMQYKAMQA), a known U2AF1 binding partner (30). The peptide was dissolved in 1 mM EDTA and used at a final concentration of 10 nM. U2AF1 was serially diluted (2-fold) from 8000 nM to 0.488 nM in PBS (pH 7.4) supplemented with 0.1 mM TCEP and 0.02% Tween-20. Assays were performed in black, flat-bottom 96-well plates (Costar) and read using a Cytation5 plate reader (excitation: 485 nm; emission: 520 nm).

For the U2AF1–SRSF1 interaction assay, endogenous cysteines C16 and C148 in SRSF1 were mutated to serine, and T248 was mutated to cysteine for site-specific labeling with Alexa Fluor 488 C5-maleimide. Labeled SRSF1 (10 nM) was titrated with U2AF1 ranging from 8000 nM to 1.95 nM (2-fold dilutions) using the same buffer and plate conditions. Fluorescence was measured at 520 nm (emission) with 485 nm excitation. All assays were performed in triplicate.

Binding data were fit to the following equation:

(1) $$F_{P}=F_{\min}+\left(F_{\max}-F_{\min}\right)\left\{\frac{\left(\left[P_{T}\right]+\left[L_{T}\right]+K_{D}\right)-{\left\{{\left(\left[P_{T}\right]+\left[L_{T}\right]+K_{D}\right)}^{2}-4\left[P_{T}\right]\left[L_{T}\right]\right\}}^{0.5}}{2\left[L_{T}\right]}\right\}$$

### Fluorescence intensity ratio between tyrosine and tryptophan

FirbY-W measurements were performed using a Varian Cary Eclipse fluorometer at 295 K, as previously described (33). Briefly, 600 μl of protein (15 μM) was buffer exchanged into 400 mM Arg/Glu (pH 6.0) supplemented with 0.5 mM TCEP, centrifuged at 14,000*g* for 10 min at 4 °C, and transferred into a quartz cuvette. Fluorescence emission spectra were recorded from 280 to 400 nm using an excitation wavelength of 275 nm and slit widths of 5 nm for both excitation and emission.

Protein denaturation was performed by titrating with buffer containing 400 mM Arg/Glu (pH 6.0), 0.5 mM TCEP, and 8 M urea to achieve final urea concentrations ranging from 0 to 7 M. The emission peaks corresponding to tyrosine and tryptophan were deconvoluted using a log-normal distribution model (51, 52). Fluorescence intensity ratios (FirbY-W) were then calculated using the following equation:

(2) $$FirbY-W=\frac{D*\exp\left(\frac{mx-\Delta G}{RT}\right)+N}{\exp\left(\frac{mx-\Delta G}{RT}\right)+1}$$

Where *x* is the urea concentration, *N* and *D* are the baselines for the native and denatured states, *m* is the urea m-value, and *ΔG*, *R*, and *T* represent the unfolding free energy, gas constant, and temperature, respectively.

### Pull-down assays

Purified U2AF1 (1 μl of 100 μM) was incubated with 10 μl of Ni-NTA agarose beads at 4 °C. Beads were washed with 100 μl of 10 mM Tris-HCl (pH 7.4), 150 mM NaCl, 1% Triton X-100, 1 mM PMSF, protease inhibitor cocktail, and 200 mM Arg/Glu and subsequently incubated with either 1.34 μl of 1 μM phosphorylated full-length SRSF1 or HeLa cell lysate (ATCC product, prepared from 0.8 million cells) overnight at 4 °C.

After binding, the beads were collected by centrifugation at 8500*g* for 90 s and washed three times with 1 ml of the same buffer. Bound proteins were eluted using 500 mM imidazole, 20 mM Tris-HCl (pH 8.0), 150 mM NaCl, 0.02% Tween-20, 1 mM PMSF, and protease inhibitors. Samples were analyzed by SDS-PAGE followed by Western blotting.

Proteins were transferred to PVDF membranes at 4 °C with a current of 400 mA for 1 h, then blocked with 5% BSA for 2 h at room temperature. Blots were incubated overnight at 4 °C with primary antibodies: anti-SUMO polyclonal antibody (1:3000, Cat# 200-401-428) for detecting His-SUMO-U2AF1 and anti-SF2 antibody (1:1000, ab38017) for detecting phosphorylated SRSF1. Membranes were then washed three times with 15 ml of PBST buffer and incubated with HRP-conjugated secondary antibodies: anti-rabbit IgG (1:10,000 for U2AF1, 1:3000 for SRSF1). Signals were visualized using SuperSignal West Pico PLUS chemiluminescent substrate.

### *In vitro* phosphorylation of U2AF1 and MS

Two hundreds μM of His-SUMO-tagged U2AF1 RS domain (residues 181–240, G210 C mutant) was phosphorylated *in vitro* by incubation with 4 μM CLK1 (residues 142–484) and SRPK1 in 1 ml of phosphorylation buffer (50 mM Tris-HCl, pH 7.5, 150 mM NaCl, 10 mM MgCl_2_, 2 mM ATP, and 0.2 mM TCEP) at 30 °C for 1 h. The reaction was quenched with 1 ml of 4 M urea and 15 mM EDTA. The sample was subsequently purified using a Mono S HR 5/5 column to separate phosphorylated U2AF1 from the kinases. One micromolar, 10 μl of the purified phosphorylated protein was analyzed by MS. Samples were diluted into 0.1% formic acid and separated on Waters BEH SEC column 200A, 2.1 x 150 mm column using 0.1% formic acid at a flow rate of 70 μl/min. The eluate of the column was connected inline to the ESI source of a Waters Synapt G2-S(i). Data were collected under MassLynx (Waters) in positive ion, resolution mode. Spectra were summed across the elution peak, and the charge state series was fit using Waters MaxEnt1.

### Purification of U2AF1 from mammalian cells

The human U2AF1 gene was cloned into the pcDNA4-myc-His-A vector and transfected into 293FT cells grown in DMEM supplemented with 10% FBS and 0.4 mg/ml G418. A total of 40 million cells were harvested and lysed in 6 M guanidinium-HCl, 20 mM Tris-HCl (pH 7.5), 25 mM imidazole, 1 mM TCEP, 1% Triton X-100, and 1 mM PMSF.

Lysates were heated at 95 °C for 1 min, sonicated three times on ice, and clarified by centrifugation. The supernatant was incubated with 10 μl of Ni-NTA beads prewashed with lysis buffer. After rotating for 2 h at 4 °C, beads were washed three times with 0.5 ml of wash buffer (same as lysis buffer).

For benzonase treatment, 1 ml of benzonase cleavage buffer (4 M urea, 1 mM MgCl_2_, 10 mM Tris-HCl pH 7.5, 0.2 mM TCEP, and 1 μl benzonase; Ref#: 71,205–3) was added to the beads and incubated at room temperature for 30 min. Beads were washed with 6 M urea wash buffer and then with Glu-C buffer (150 mM ammonium bicarbonate, pH 8). Glu-C digestion was initiated by adding 1 μl Glu-C protease (Ref#: 90,054) in 50 μl Glu-C buffer, and the reaction was incubated overnight at 37 °C. The supernatant containing the peptide digest was collected and analyzed by MS.

### LC-MS/MS and protein identification

For LC-MS/MS analysis, an Ultimate 3000 RSLCnano system coupled to an Orbitrap Eclipse MS *via* a FLEX nano-electrospray source and a FAIMS Pro Interface (all from Thermo Scientific) were employed. Mobile phase A was 0.1% (v/v) formic acid in LC-MS grade water, and mobile phase B was 0.1% (v/v) formic acid in acetonitrile. The peptides were resuspended in mobile phase A and first loaded onto a trap column (PepMap100 C18, 300 μm × 2 mm, 5 μm; Thermo Scientific), followed by separation on an analytical column (PepMap100 C18, 250 mm × 75 μm i.d., 3 μm; Thermo Scientific). A linear LC gradient with a flow rate of 250 nl/min was applied from 1% to 25% mobile phase B over 125 min, followed by an increase to 32% mobile phase B over 10 min. The column was washed with 95% mobile phase B for 5 min, followed by equilibration with mobile phase A for 15 min. For the ion source settings, the spray voltage was set to 1.7 kV, funnel RF level at 30%, and heated capillary temperature at 275 °C. For FAIMS settings, the electrode temperatures were set to 100 °C, FAIMS carrier gas N2 flow was 4.6 L/min, asymmetric waveform with DV was −5000 V, entrance plate voltage was 250 V, and CVs were set to −40, −55, and −75 V. The MS data were acquired in Orbitrap at 60,000 resolution, followed by MS/MS acquisition of the 10 most intense precursors for 1 s. The MS1 scan range was set to 375 to 1600 m/z, AGC target was set to Standard, and the maximum injection time mode was set to Auto. For MS2 analysis, precursors with charge states 2 to 5 were selected. The isolation mode was Quadrupole, collision was by HCD, and collision energy was 30%. The Orbitrap was set to detect MS2 fragments at 15,000 resolution with standard AGC target, dynamic maximum injection time, and a 1.6 m/z isolation window. Dynamic exclusion was set to 30 s. Monoisotopic precursor selection was set to Peptide.

Proteome Discoverer and Sequest HT (version 2.5; Thermo Scientific) were employed for protein identification. The following parameters were applied: 10 ppm and 0.02 Da mass tolerances for precursor and fragments, respectively; either LysC or GluC as enzyme with two missed cleavage sites; protein N-terminal acetylation, methionine oxidation, and phosphorylation (S/T/Y) as variable modifications; and cysteine carbamidomethylation as a fixed modification. Peptide length was set to at least seven amino acids. Percolator was used to calculate the false discovery rate, and 1% was chosen for protein identifications.

### *In vitro* phosphorylation of SRSF1

Full-length SRSF1 and the RS domain of SRSF1 were purified using the previously reported procedures (28, 29, 53). Purified full-length SRSF1 C16/148S T248 C (600 μl at 200 μM) was incubated with CLK1 (12 μl at 100 μM) and SRPK1 (60 μl at 22 μM) in 4 ml of phosphorylation buffer containing 50 mM Tris-HCl (pH 7.5), 10 mM MgCl_2_, 5 mM ATP, 150 mM NaCl, 0.2 mM TCEP, and 200 mM Arg/Glu. The reaction was carried out at 30 °C overnight and quenched by the addition of 1 ml of 500 mM EDTA. To maintain protein solubility, the total volume was adjusted to 10 ml with 1 M Arg/Glu. The sample was concentrated to 1 ml using a 10 kDa molecular weight cutoff centrifugal filter at 4 °C and 4000 RPM, then diluted to 10 ml with buffer A (20 mM Tris-HCl, pH 7.5, 1 mM EDTA, 0.4 mM TCEP). The diluted sample was loaded onto a 1 ml Mono Q 5/50 Gl column, and phosphorylated SRSF1 was purified by gradient elution using buffer B (20 mM Tris-HCl, pH 7.5, 1 mM EDTA, 0.4 mM TCEP, 1 M NaCl). His-SUMO-tagged SRSF1 RS domain (residues 199–248, T248 C) was phosphorylated and purified using the same procedure.

### MD simulation and analysis

MD simulations were conducted using AMBER in multiple stages: minimization, heating, equilibration, and production for complexes of U1-70K BAD1: phosphorylated SRSF1 RS, U2AF1 RS: phosphorylated SRSF1 RS, and SRSF1 RS: phosphorylated SRSF1 RS, as well as corresponding monomeric molecules. To estimate errors, three distinct starting conformations were used for each system. Systems were solvated in OPC water and 150 mM Na^+^ or Cl^-^ with a 10 Å cutoff. Additional Na^+^ or Cl^-^ ions were added to neutralize the net charge. Peptides were modeled using the FF19SB and phosaa19SB force fields (54, 55). Each system underwent energy minimization with 2500 steps of steepest descent followed by 2500 steps of conjugate gradient. This was followed by 50 ps of heating from 0 K to 300 K in NVT ensemble and 50 ps of equilibration in NPT ensemble, with positional restrains on water and ions. Production MD was carried out for 360 to 520 ns in the unstrained NPT using a Langevin thermostat, a 2-fs time step, and trajectory snapshots saved every 150 ps.

RMSDs were calculated for backbone to confirm the systems reach equilibration. Hydrogen bond, salt bridges, and stacking interactions, including π-π stacking, cation-π stacking, and cation-cation stacking (Arg-Arg), were analyzed. For salt bridges, arginine and lysine side chains were used for donors, and aspartate, glutamate, and phosphoserine side chains were used for acceptors. Stacking interactions were identified using a distance cutoff of 4.5 Å and an angle threshold of 20°.

## Data availability

All data are contained within the manuscript.

## Supporting information

This article contains supporting information.

## Conflict of interest

The authors declare that they have no conflicts of interest with the contents of this article.
