# Supplementary material for: Phosphorylation-dependent regulation of serine/arginine-rich proteins and U2AF1 interactions in early spliceosome assembly
Source: J Biol Chem. 2026 Feb 2;302(3):111222. doi: 10.1016/j.jbc.2026.111222 (PMC13080583; doi:10.1016/j.jbc.2026.111222)
Supplement: Supplemental material [file mmc2.docx]

**Molecular Basis of RS Domain-Mediated Interactions in Early-Stage Spliceosome Assembly: Insights from U2AF1 and SRSF1**

Zihan Zhang^1^, Puspa Kunwar^1^, Yanbao Yu^2^, Peter Prevelige^3^, Jun Zhang^1,^*

^1^Department of Chemistry, University of Alabama at Birmingham, Birmingham, AL, 35294, USA

^2^Department of Chemistry and Biochemistry, University of Delaware, Newark, DE, 19716, USA

^3^Department of Microbiology, University of Alabama at Birmingham, Birmingham, AL, 35294, USA

*To whom correspondence should be addressed. Tel: +1 205 934 2139; Fax: +1 205 934 2543; Email: zhanguab@uab.edu

Key words: U2AF1, U2AF35, SRSF1, RNA splicing, phosphorylation

**Table S1. Mass spectrometry analysis of U2AF1 samples purified from 293FT cells.**

The table summarizes protein identification results. Columns include the confidence score of protein identification, UniProt accession number, protein description, number of peptide-spectrum matches (PSMs), number of unique peptides identified, sequence coverage (%), molecular weight (MW), isoelectric point (pI), amino acid (AA) length, and estimated probability of sample contamination.


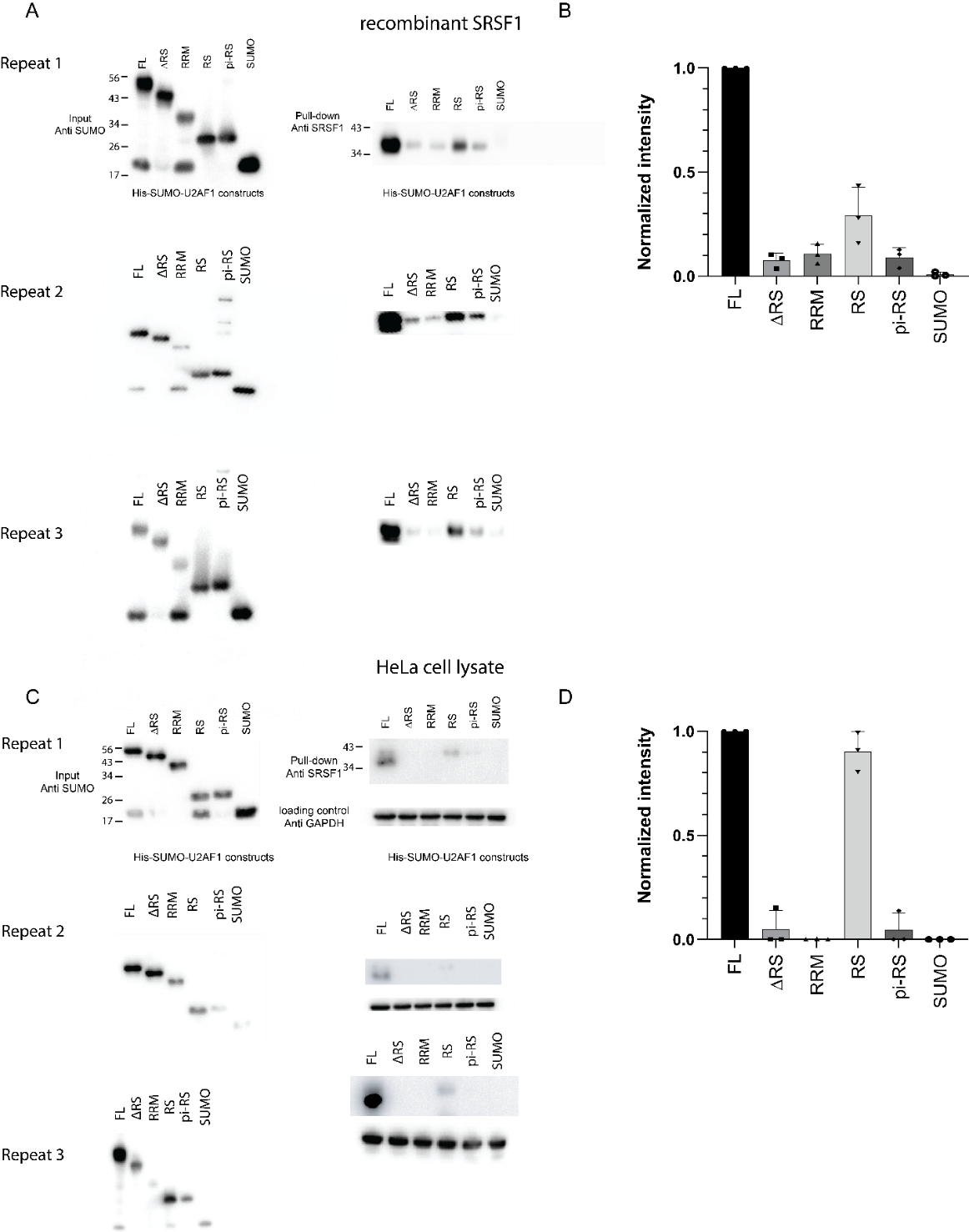


Figure S1: The RS tail of U2AF1 mediates its interaction with phosphorylated SRSF1 (pi-SRSF1). (A) Western blotting images of three replicated pulldown assays using recombinant phosphorylated SRSF1. His-SUMO tagged U2AF1 were immobilized on Ni-NTA bead as baits. Hyper-phosphorylated SRSF1 purified from E. coli was used as the prey. Sumo-specific and SRSF1-specific antibodies were used to detect the input U2AF1 and pull-down SRSF1. (B) The bar graph demonstrates the band intensities indicated by the rectangular boxes. (C) Western blotting images of three replicated pulldown assays using HeLa cell lysate. HeLa cell lysate was used as the prey. GAPDH-specific antibody was used to ensure similar amounts of cells were used. All pull-down assays were performed three times, and the standard deviation was estimated from these replicates. (D) The bar graph demonstrates the band intensities indicated by the rectangular boxes. The positions of molecular markers (KDa) are shown at the left side.


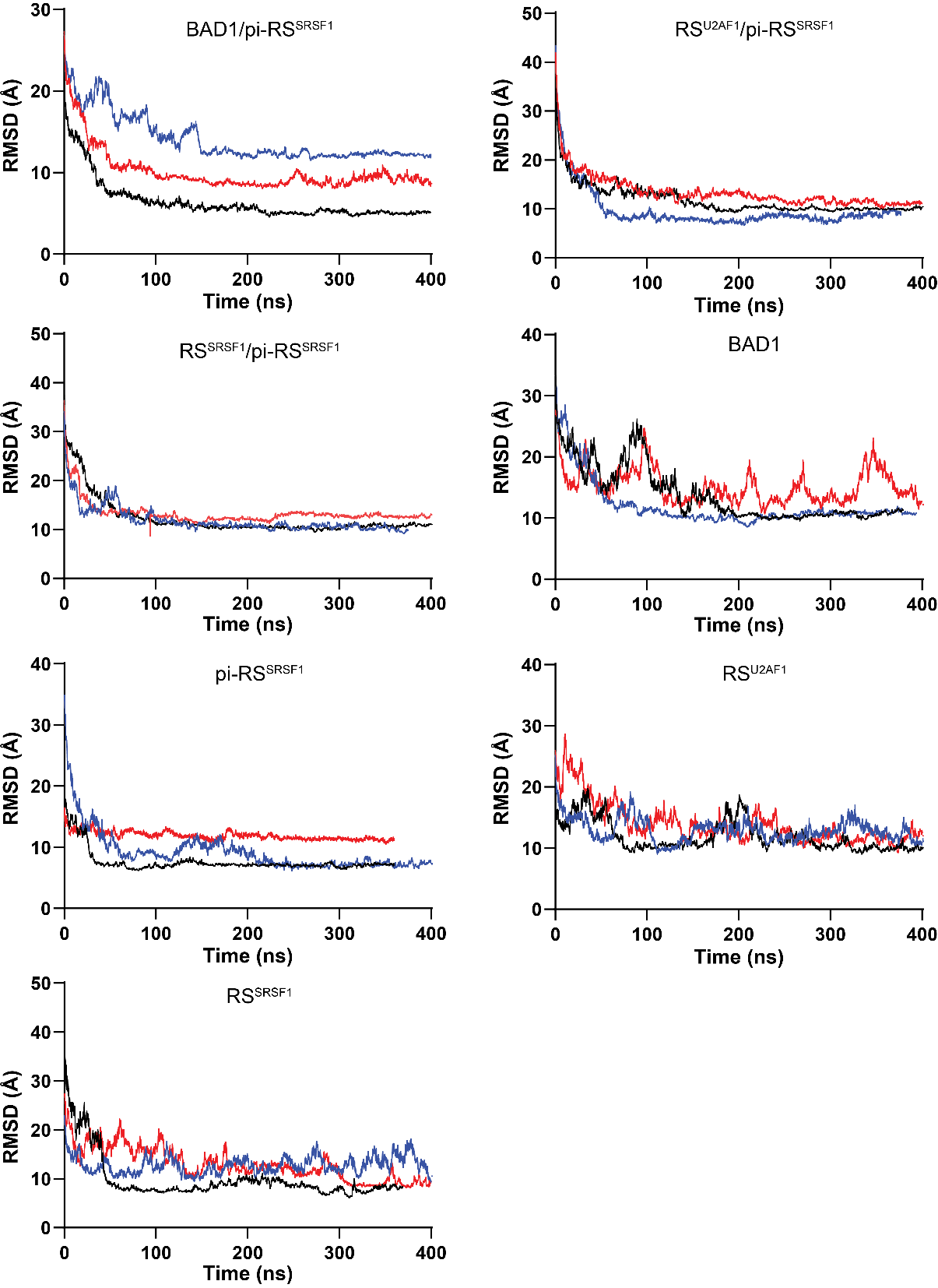


Figure S2. RMSD values between the average coordinate and coordinates of all atoms of the peptides over time in the MD simulations.
